# Testing the feasibility of deep learning approaches to enhance monitoring of marine macroinvertebrates: Insights from a case study using the gastropod *Peringia ulvae*

**DOI:** 10.1007/s10661-026-15682-7

**Published:** 2026-07-14

**Authors:** Israel Campero Jurado, Hannah S. Earp, Joaquin Vanschoren, Heather Sugden

**Affiliations:** 1 Mathematics and Computer Science, Eindhoven University of Technology, Eindhoven, 5612 AZ Noord-Brabant The Netherlands; 2 The Dove Marine Laboratory, School of Natural and Environmental Sciences, Newcastle University, Cullercoats, North Shields, NE30 4PZ UK; 3 Institute of Marine Research, 4817 His, Norway

**Keywords:** Ecological monitoring, Automated image analysis, Object counting, Machine learning, Artificial intelligence, Method development

## Abstract

Coastal ecosystems, while vital for their ecosystem services, face growing threats from climate change and human activities. Traditional methods for monitoring these systems, such as morphological species identification and laboratory analysis, are time-consuming and resource-intensive, restricting their scalability. Recent advances in Deep Learning have demonstrated significant potential for automating organism identification and counting from images, streamlining ecological research efforts. Using a common marine gastropod, *Peringia ulvae*, as a model species, we evaluate the potential for state-of-the-art computer vision methods incorporating self-supervised and few-shot learning techniques, to quantify this marine macroinvertebrate. We tested five machine learning models (CounTR, Segment Anything, Training-Free Object Counting, Grounding DINO, DeepDataSpace) across imaging devices, organism aggregation levels, and compared the performance across models and with human counts to determine accuracy and robustness. Using high-resolution images from a DSLR camera, we found that CounTR and Deep Data Space excelled in scenarios where *P. ulvae* were aggregated as opposed to spaced. However, model accuracy plateaued at ***∼***400 individuals per image, emphasizing the need for standardized imaging protocols. We also explored the strengths and limitations of computer vision–based approaches compared to traditional laboratory methods. Although computer vision is not a replacement for traditional laboratory techniques, it can offer a scalable alternative for processing samples associated with ecological monitoring programs if models are trained appropriately. This study highlights the potential of artificial intelligence to transform ecosystem research while pinpointing areas for development, such as adapting models for mixed-species samples and considering associated detritus.

## Introduction

Coastal ecosystems are diverse and dynamic, with complex interactions between organisms and environmental variables underpinning the valuable ecosystem services they provide (Costanza et al., 1997, 2014; Worm et al., 2006). Located at the interface between terrestrial and marine environments, means however, that coastal systems are subject to increasing pressures including climate change and anthropogenic activities, both of which vary in distribution and intensity across spatial and temporal scales. In many areas, these stressors have had profound, synergistic impacts on the structure and functioning of ecosystems and the services they provide (Halpern et al., 2008; Poloczanska et al., 2013; Wernberg et al., 2024). Although to fully understand the influence of such stressors on key ecosystems, data covering extensive spatio-temporal scales are required (Dafforn et al., 2015). Traditional methods to study coastal ecosystems (e.g. field sampling, morphological species identification, and laboratory processing) are essential to understand the influence of environmental change. However, these methods are often time-consuming and costly, with a low potential for up-scaling, meaning they can limit the resolution of data sets and delay decisions aiming to mitigate disturbances and protect key ecosystems (Danovaro et al., 2016). As such, there is a growing interest in developing techniques that can enhance the efficiency of collecting, processing and analyzing field samples. Recent technological advances, particularly in Artificial Intelligence (AI), are transforming traditional research methods by enhancing the scale and resolution of datasets. AI, especially through Deep Learning—a machine learning subset using deep neural networks—excels in analyzing large datasets, such as in image and speech recognition. Developments in computer vision that leverage AI and Deep Learning to train computers to interpret and obtain meaningful data from media inputs have become essential across domains including object detection (Carion et al., 2020; Dai et al., 2017), image classification (Hu et al., 2018; Woo et al., 2018), face recognition (Wang et al., 2020; Yang et al., 2017), semantic segmentation (Fu et al., 2019; Yuan et al., 2018), and beyond.

Accurate and efficient object counting holds immense value, enabling informed decision-making, resource allocation, and trend analysis. Within the realm of object counting, the counting of organisms in ecological studies is well understood for easily distinguishable species, but challenges and opportunities exist for its use in identifying and counting cryptic, morphologically similar, and/or microscopic organisms, despite being important indicators of ecosystem health (Gibert et al., 2022; Pinho et al., 2022; Zare et al., 2022). While object counting in laboratory-controlled environments or with distinct objects may seem straightforward, the counting of such organisms can be challenging due to variations in size, shape, color, orientation, occlusions, and level of aggregation. In this work, we implement state-of-the-art computer vision methods that combine self-supervised and few-shot learning into object counting. We compare five methods to investigate their efficacy in detecting and enumerating a single marine macroinvertebrate, the mud snail *Peringia ulvae*, to evaluate the potential of using computer vision methods for such analyses, with the aim of scaling-up to multi-species samples through future work. Through this comparative analysis, we aim to identify the most effective approach for quantifying *P. ulvae* abundance and to gain insights into the broader implications and limitations of object counting methods in ecological research. The study is divided into four sections:

1. To test the feasibility of a computer vision algorithm in enumerating *P. ulvae* in images collected using three different digital devices.
2. To determine if the level of aggregation of *P. ulvae* individuals influences the accuracy and carrying capacity of different deep-learning techniques.
3. To examine the variance and robustness of digital versus human object counting approaches.
4. To highlight the advantages and limitations of deep learning techniques over traditional laboratory approaches.

## Materials and methods

### Sample collection

Between July and September 2022, samples of opportunistic green macroalgae (*Ulva* spp.) were collected from fixed-point quadrats around the perimeter of Budle Bay, an intertidal mudflat along the northeast coast of the United Kingdom (UK; 55°36′41.3″N, 1°46′10.7″W) as part of a long-term ecological monitoring project. Macroalgal samples were returned to the laboratory where each sample was washed over two buckets of freshwater to remove all epibiota. The freshwater buckets were then rinsed over a 0.5-mm sieve to collect all epibiotes and these were placed in pots and preserved using 70% Industrial Methlylated Spirit (IMS). Epibiotes were pre-sorted to the lowest taxonomic resolution possible and enumerated. Epibiota samples were often dominated by the mud snail *P. ulvae* (Pennant, 1777), a common gastropod mollusc in estuarine areas that can reach densities of over 100,000 ind. *m*^*−*2^ (Sola, 1996).

### Comparing across digital devices

**Fig. 1:**
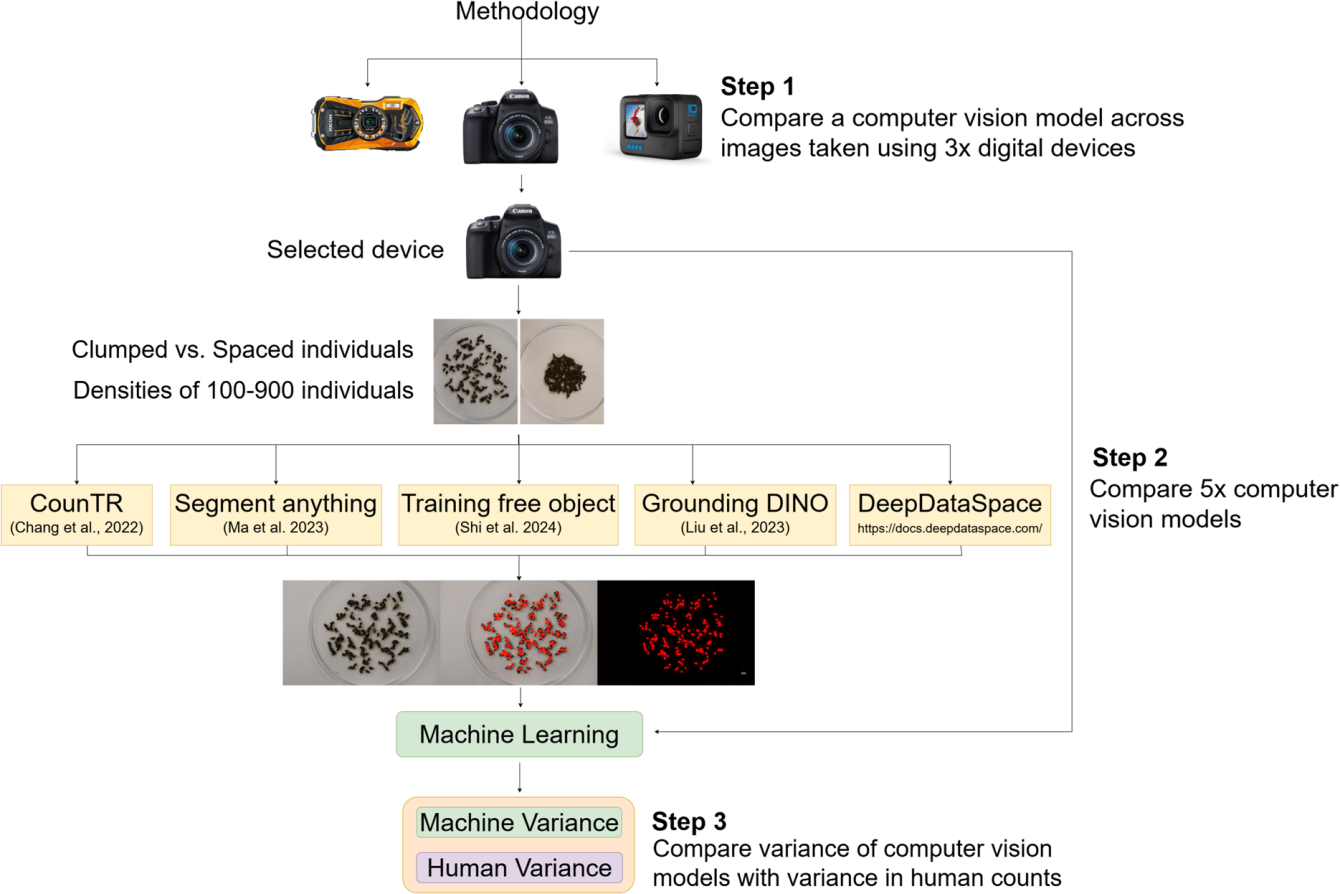
Methodology flowchart including digital device selection, computer vision models and variance analyses

In the first instance, ten samples of *P. ulvae* of known densities (ranging from 64 to 2424 individuals) were randomly selected. Each sample was placed on a petri-dish with a white background and photographed using three digital camera types; (1) a RICOH WG30 digital camera (PP), (2) a Cannon EOS 850D DSLR camera (DSLR), and (3) a GoPro 10 (GoPro) (Fig. 1). Each petri-dish was rotated slightly and rephotographed twice more to give three photos per sample and a library of 90 images.

These images were analyzed through CounTR (Chang et al., 2022), a computational model designed for versatile visual object counting across diverse semantic categories in images. This model is a transformer-based architecture using attention mechanisms Vaswani et al. (2017) to analyze relationships between image patches and user provided exemplars. The model employs a dual stage training process, commencing with self-supervised learning and refining through fine-tuning for optimal performance.

### Accuracy across densities and levels of aggregation

*P. ulvae* (also referred to as’exemplars’) were separated into batches of known quantities ranging from 100 to 900 individuals, with incremental increases of 100 individuals. Each batch was placed in a petri-dish with a white background and photographed twice; once with all individuals pushed together into a central aggregation or’clump’, and once with individuals spaced out so there was a degree of white space between individuals (Fig. 1). Photos were taken in one orientation using a DSLR camera to give a library of 18 images.

Five different computational models were run on this image library, and their performance was compared (Fig. 1). The first model was CounTR (Chang et al., 2022), which was followed by Segment Anything (SAM) (Ma et al., 2023), a model developed by Meta AI to do image segmentation. The third model was Training-free Object Counting with Prompts (Shi et al., 2024), which leverages SAM using a segmentation model that identifies individual objects based on specified prompts including text inputs, boxes or points. Grounding DINO (Liu et al., 2023), an open-set object detector that leverages a transformer-based architecture with grounded pre-training to detect objects based on text inputs was the fourth model used. The final model was DeepDataSpace, an open-source framework focused on dataset tools for dataset visualization, labelling and counting, https://github.com/IDEA-Research/deepdataspace. Three of the models, SAM, Grounded DINO, and Training-free Object Counting with Prompts, count objects based on text inputs, and in these cases, the prompt’snails’ was used. CounTR and DeepDataSpace require annotated images with coordinates defining rectangles of pixels containing examples of the object of interest using two sets of coordinates: (*x*_1_*, y*_1_) for the lower left corner and (*x*_2_*, y*_2_) for the upper right corner.

### Understanding variance across techniques

Using images containing 100–400 clumped and spaced *P. ulvae* from the image library generated above, model prompts and coordinates associated with the images were systematically varied to investigate how the choice of exemplars, including their diversity and representativeness can influence the performance of the computational models (Fig. 1). For models that require coordinates to construct a density map (i.e. CounTR and DeepDataSpace), a new set of annotated images/coordinates were provided. While for models requiring text prompts (i.e. SAM, Grounding DINO and Training-free object counting), a different set of prompts was defined: ‘snails’, ‘tiny snails’, ‘water snails’, ‘aquatic snails’, ‘stream snails’, ‘wetland snails’, ‘sea snails’, ‘marsh snails’, ‘bay snails’, and ‘underwater snails’ were used. Each image was analyzed ten times by each model using a different combination of prompts/coordinates per iteration. To estimate the variance in human counts, a single marine scientist independently counted *P. ulvae* within the same image library, with each image analyzed six times using ImageJ (Schneider et al., 2012).

### Data analysis

To compare CounTR counts of *P. ulvae* across digital devices and photo orientations with human counts, a two-way analysis of variance (ANOVA) was conducted with counting method (four levels, fixed), sample orientation (three levels, fixed) and their interactions given as factors. To compare variance in computational models versus human counts of *P. ulvae* in images of different densities and levels of aggregation, a two-way ANOVA was undertaken with counting method (six levels, fixed), aggregation level (two levels, fixed) and their interactions given as factors. Where significant differences were detected (*p* < 0*.*05), Tukey’s HSD pairwise post hoc analyses were conducted. All analyses were undertaken using Jamovi software [Version 2.3.28] (The jamovi project, 2022).

### Digital versus traditional techniques

The benefits and challenges of machine learning versus traditional laboratory identification and enumeration techniques were arbitrarily assessed taking into account equipment requirements, the training and experience/expertise needed, the time to enumerate a sample of *∼*100 *P. ulvae*, method accuracy and their potential to be incorporated into research efforts spanning greater spatio-temporal scales.

## Results

### Comparing across digital devices

**Fig. 2:**
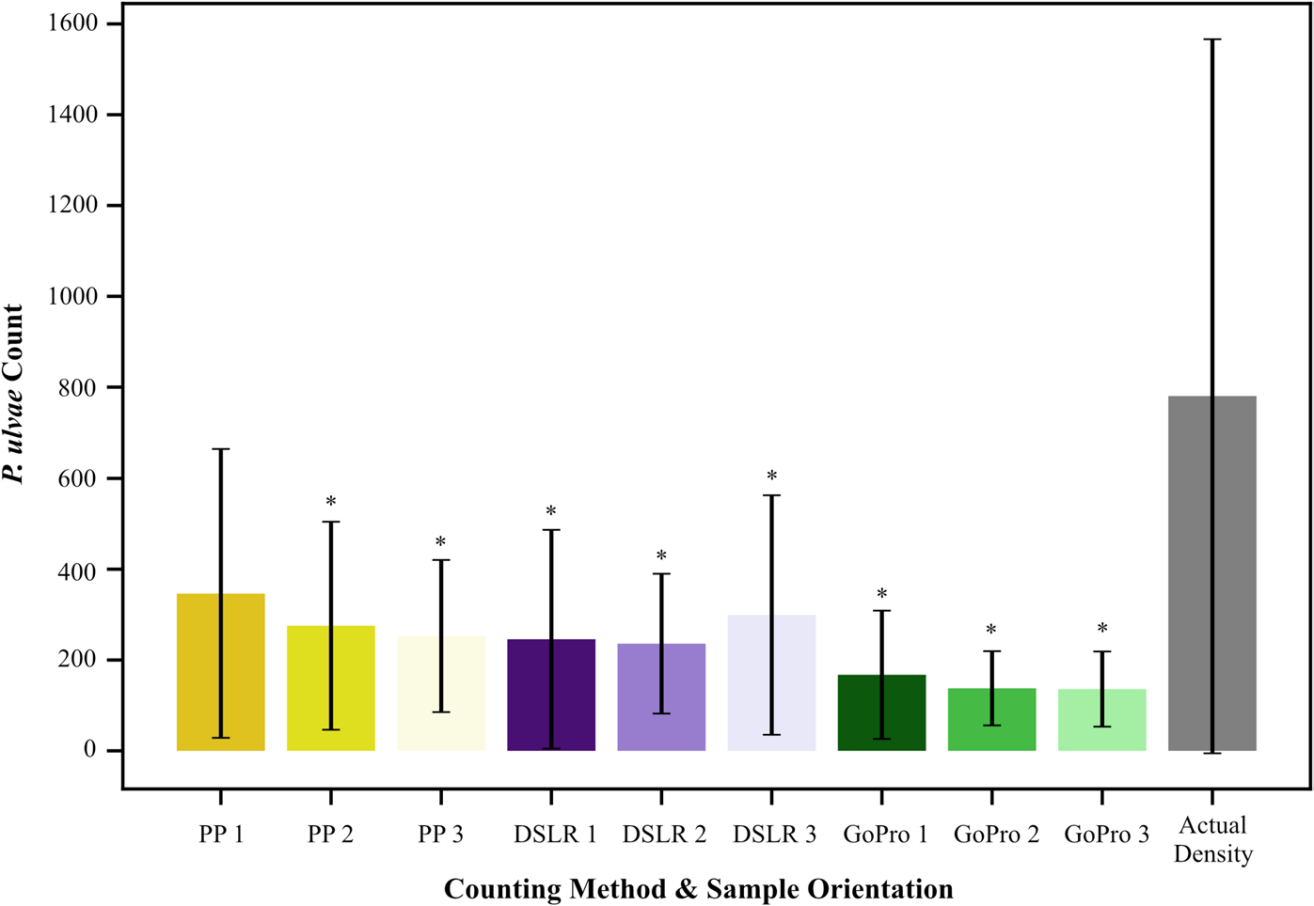
Mean (± 1 standard deviation) *P. ulvae* counts obtained using CounTR analyses of images (*n* = 10) from three different digital devices (DSLR, GoPro, PP) and sample orientations (1–3) compared to the actual density. Significant pairwise differences between digital devices and image orientations compared to human counts are shown with asterisks

There was a significant interaction between *P. ulvae* counts obtained by the different counting methods (i.e. CounTR of DSLR, PP and GoPro images, and actual density of the sample) and the sample orientation (*F*_9*,*90_ = 3*.*379, *p* < 0*.*01). Post hoc analyses revealed that these differences were most pronounced between actual density and all digital devices and sample orientations, with the exception of PP 1 (Fig. 2). A significant difference between the different counting methods alone was also observed (*F*_3*,*90_ = 4*.*044, *p* < 0*.*05), with significantly greater actual density observed compared to CounTR analyses of images from each of the three digital devices. As CounTR counts of *P. ulvae* did not significantly differ between the three digital devices, the DSLR was selected for use in further trials owing to its ease of use and greater photo quality compared to the PP and GoPro. There was also a significant difference between CounTR counts across the different sample orientations; however, as the orientations were haphazardly produced and thus not replicable, no further analyses of this factor were undertaken.

### Accuracy across densities and levels of aggregation

**Fig. 3:**
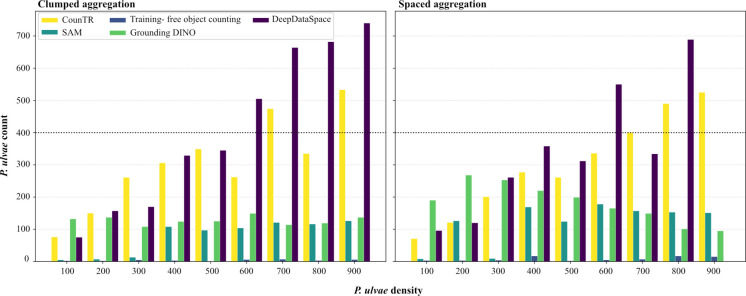
Total counting accuracy of five computer vision models using a single prompt/coordinate, across images (taken using a DSLR camera) of known *P. ulvae* densities (100–900) in clumped and spaced aggregations. Hashed line represents the threshold of 400 individuals where model performance declined

Deep Data Space consistently achieved the most accurate counts across the images, although its performance showed noticeable declines for images of spaced *P. ulvae* towards the highest aggregation levels, particularly 700 and 900 individuals (Fig. 3). Both CounTR and Deep Data Space exhibited the most stable performances up to 400 individuals in both the clumped and spaced images (Fig. 3). There was an impasse in the performance of most models (excluding SAM) after 400 individuals. Based on this observation, a maximum of 400 *P. ulvae* was selected as the threshold for further analysis of aggregation levels and their associated variance, to investigate the robustness of model performance. In general, the performance of SAM, Training-free Object Counting and Grounding Dino was fairly inconsistent, and they provided counts that were considerably lower than the number of individuals present in the photos.

### Understanding variance across techniques

**Fig. 4:**
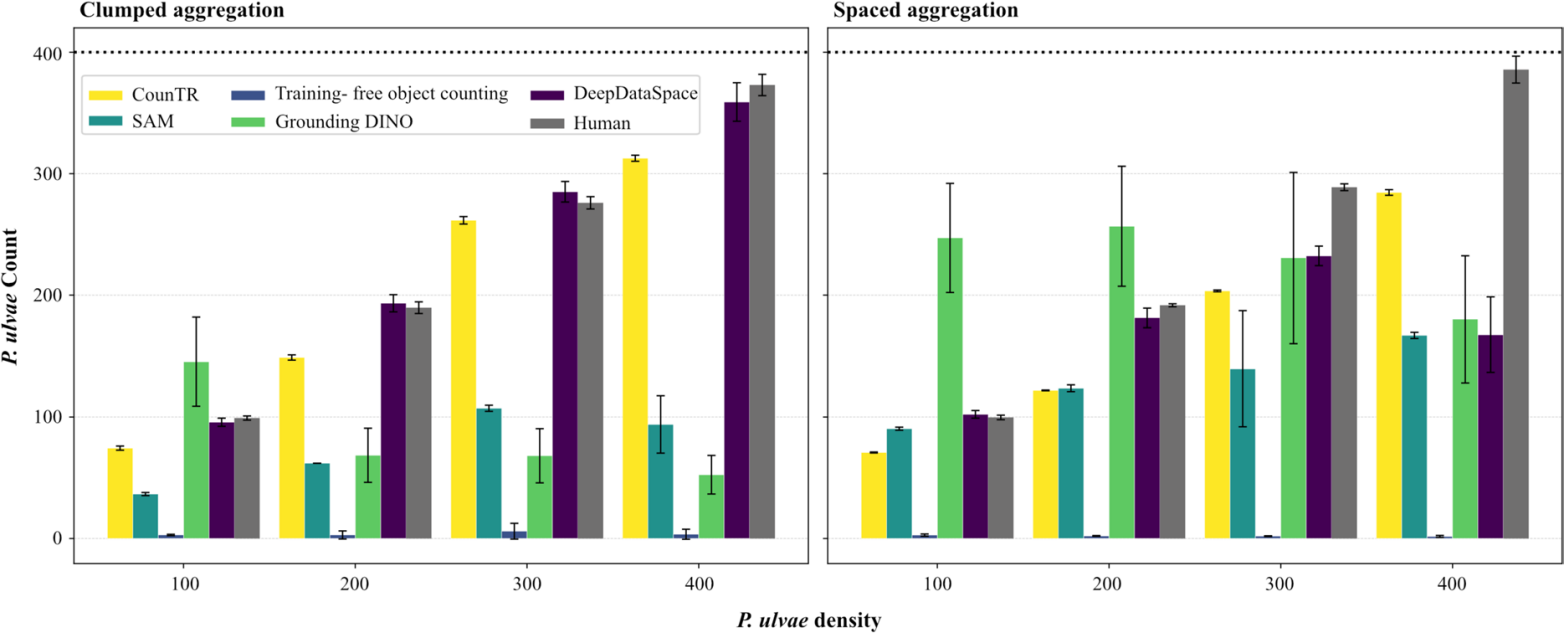
Mean (± 1 standard deviation) *P. ulvae* density counts generated by five computer vision models for images (taken using a DSLR camera) of known *P. ulvae* densities (100–400) in clumped and spaced aggregations. Each image was analyzed ten times by each model using a different combination of prompts/coordinates per iteration, and six times by a single scientist. Hashed line represents the threshold of 400 individuals where model performance declined

There was no significant interaction between the method used to count *P. ulvae* (i.e. computer vision models vs human counts) and the level of *P. ulvae* aggregation (i.e. clumped vs spaced aggregation) (*F*_5*,*36_ = 1*.*19, *p* = 0*.*334). However, there was a significant difference in the counts obtained using the different counting methods alone (*F*_5*,*36_ = 9*.*51, *p* < 0*.*001) (Fig. 4). Pairwise post hoc analysis revealed that SAM and Training-free Object Counting significantly underperformed relative to human counts, but there was no difference between human counts and those obtained using Deep Data Space, CounTR and Grounding DINO (Fig. 4). Deep Data Space and CounTR counts in particular, aligned well with human counts, especially in images where *P. ulvae* were clumped together, while Grounding DINO had a tendency to over count *P. ulvae* in spaced images and under count in clumped images (Fig. 4).

### Digital versus traditional counting techniques

The strengths and limitations of traditional laboratory techniques and deep learning approaches for identifying and enumerating monospecific marine macroinvertebrate samples were assessed (Table 1). Regarding the set-up, traditional laboratory techniques require laboratory and taxonomic expertise but do not necessitate digital or computing expertise, which is essential for deep learning approaches. The laboratory techniques rely on microscopes and taxonomic knowledge, while deep learning approaches require both a camera and a computer, demonstrating a clear divide in the skill sets needed for each technique. Regarding their application, traditional laboratory techniques are time-intensive, while deep learning approaches offer significant time efficiency. Both methods demonstrate comparable accuracy for monospecific samples, but deep learning techniques exhibit a notable advantage in their potential to increase the spatio-temporal scope of research. This capability is particularly beneficial for large-scale studies where traditional methods may be limited by time and labor constraints.

**Table 1:**
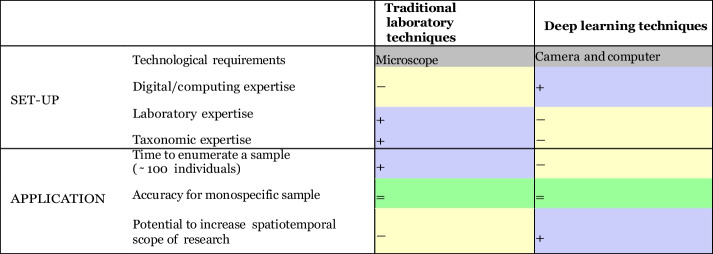
Strengths and limitations of traditional laboratory techniques compared to computer vision approaches for identifying and enumerating monospecific marine macroinvertebrate samples. (+) indicates a greater requirement, while (−) and (=) indicate lower and equal requirements respectively

## Discussion

Deep learning approaches are becoming increasingly common place in scientific research, particularly to identify and enumerate organisms within images (Farjon et al., 2023; Singh et al., 2023). Here we advanced the use of computer vision models, using them to classify and count microscopic a common marine macroinvertebrate, *P. ulvae*, in images. We found that sample orientation, organism density and level of aggregation, and the choice of computer vision model all influenced digital count accuracy, with values obtained by computer models generally lower than those obtained by human counts — highlighting the importance of benchmarking procedures. We also assessed the strengths and limitations of computer vision techniques compared to traditional laboratory procedures for analyzing macroinvertebrate samples. Collectively, our results revealed that while not a substitute for traditional laboratory procedures, computer vision offers a means of increasing the scope of research efforts, but before being incorporated into research endeavors consideration must be given to the sample presentation and trials comparing a selection of computer vision models is imperative to identify the most appropriate model for the samples being analyzed.

While we found little difference in counts generated from images captured using three different digital devices, suggesting that cheaper and simpler cameras (i.e. PP, GoPro) may be as effective as professional cameras (i.e. DSLR), we continued to use the DSLR throughout the research as it provided the highest quality images. High-resolution images can provide greater detail on organisms including their shape, size, and texture, all of which can improve the accuracy of counting algorithms. For example, a study using deep learning with self-supervision and uncertainty regularization to count fish in underwater images found that model performance was greater on high-resolution images (Tarling et al., 2022). As technology continues to advance, future efforts may consider exploring the use of mobile phone cameras that are widely owned and therefore could easily be incorporated into research efforts (including citizen science), easing image sharing/uploads that can be done directly from the device.

For count accuracy across *P. ulvae* densities and levels of aggregations, CounTR and Deep Data Space yielded the most accurate values, particularly in images where individuals were presented in a central aggregation/clump. CounTR is transformer-based and specializes in general visual counting. Transformer models are inherently strong in capturing global context and spatial relationships within images (Khan et al., 2022), which may aid in the recognition of clusters of similar objects more effectively than isolated or spaced objects. The strength of Deep Data Space originates from the combination of multiple state-of-the-art (SOTA) techniques, such as Grounding DINO, T-Rex2 (Jiang et al., 2024), and the recently proposed DINO-X (Ren et al., 2024). This combination allows it to leverage the unique advantages of each model therefore it excels at precise object localization and association, bringing robustness in feature extraction and representation across varying contexts (T-Rex2). This allows the model to incorporate enhanced mechanisms for fine-tuning object relationships and contextual understanding. However, Deep Data Space is not open source, which represents a challenge for broad application. The underperformance of models such as SAM and Training-free Object Counting may reflect a lack of domain adaptation, that is, no fine-tuning was performed for the current analysis, instead the models are trained on real and open-world images, for example Training-free Object used FSC147 (day-to-day photos) (Ranjan et al., 2021) and CARPK (car parking lot dataset) (Hsieh et al., 2017) datasets for evaluation. We also found that orientation of the samples, which, although not replicable, influenced the counts obtained, highlighting the need to take and analyze multiple images of each sample (where possible) and to use mean (± variance estimates) count values.

We found that the model performance tended to plateau at *≈* 400 individuals. This limitation may stem from the complexity of the models in effectively distinguishing and counting densely packed objects, as high-density images can lead to occlusion and overlapping features that challenge object detection algorithms. Transformer-based models rely heavily on global context and spatial relationships, that become less reliable as the number of objects increases, potentially causing a bottleneck in performance. Moreover, the lack of spatial context within laboratory sample preparation/presentation could influence count accuracy relative to samples photographed in field environments. Analogous challenges have been noted in studies using image-based identification systems for biological specimens, where carrying capacity appears to depend on factors such as image resolution, object size, and the model’s ability to differentiate closely spaced objects. For example, Jaballah et al. (2023)demonstrated an upper limit of 200 individuals per image when monitoring live freshwater macroinvertebrates using their MacroNet system. Similarly, Arje et al. (2020) noted high classification accuracy for invertebrate specimens in controlled laboratory settings, but their work did not extend to high-density counting scenarios. The maximum number of objects (or organisms) per image can vary greatly depending on the dataset and the specific task at hand. Some datasets, such as PerSense (Siddiqui et al., 2024), have an average count of 39 objects per image, with a total of 28,395 objects across the entire dataset. In the context of object counting tasks, the maximum number of objects per photo can influence the performance of the model. For example, if a model is trained on a dataset with a maximum of 3 objects per image, it may struggle to accurately count objects in images with more than 3 objects (Dorkenwald et al., 2024). These findings suggest that the threshold observed in our study could represent a practical carrying capacity for current deep learning models in similar ecological applications. Future work might address this limitation by developing specialized techniques for handling high-density scenarios, such as enhanced resolution models, improved feature disentanglement mechanisms, creating datasets with larger population of individuals per image or multi-scale analysis approaches.

To the best of our knowledge, the models used in this work have yet to be bench-marked against each other, although some have been compared to other models in different domains/fields (Amini-Naieni et al., 2023; Goldman et al., 2019; Shi et al., 2022; Wei et al., 2024). This study represents an important first step toward applying machine learning to the analysis of marine macroinvertebrate samples. By using *P. ulvae* as a case study, we demonstrate the feasibility of this approach and provide a benchmark of model performance for future applications. However, the use of ‘clean’, monospecific samples is not a realistic representation of field samples that often contain multiple species and sediment/detritus meaning a degree of pre-sorting (as was undertaken in this study) is required prior to any analyses of a similar nature. Additional research is required to determine the feasibility of machine learning approaches to identify and enumerate organisms in ‘mixed’ field samples, which come directly from the field with no pre-sorting. A first step in this would be to test and refine model performance using samples containing morphologically similar (e.g. two gastropod species) and distinct individuals (e.g. gastropods vs. bivalve, or hard vs. soft-bodied taxa) (Ditria et al., 2020; Xu et al., 2019). Initial research in this area has demonstrated the success of image-based identification procedures in accurately classifying (but not counting) macroinvertebrate and invertebrates (Arje et al., 2020; Høye et al., 2022). It would also be beneficial to run models on samples containing dead and/or fragmented organisms/detritus as the presence and accurate identification of these factors may pose a significant challenge to automated counting. Detritus can, at times, be mistaken for organisms while certain taxa (e.g. bivalves) may appear as living or multiple organisms if the shells have been fragmented, meaning there is potential for misidentifications and under/overestimates.

Should machine learning prove feasible for identifying and enumerating mixed macroinvertebrate samples, this will significantly reduce sample processing time and ultimately enhance the cost effectiveness of the technique. Moreover, for macrobenthic samples where organism movement is often slow, it could reduce the need to preserve specimens, allowing researchers to sieve and photograph samples directly in the field before releasing them. In this way, machine learning could reduce the need to remove and preserve samples, meaning they could be photographed in situ. Moreover, if models can be trained to identify mixed samples, the use of mobile phone photographs could increase the community involvement/citizen scientist participation in scientific research, further increasing the spatio-temporal coverage and cost-effectiveness of research efforts (Catlin-Groves, 2012; Earp & Liconti, 2020; Earp et al., 2022; Langenkamper et al., 2019; Mattingly et al., 2016; Moolna et al., 2020; Robinson et al., 2024; Terry et al., 2020; Weinstein, 2017; Willi et al., 2018).

## Conclusion

This study demonstrates the potential of machine learning approaches to enhance the efficiency and scalability of marine macroinvertebrate monitoring, highlighting that some models can be trained to correctly identify and count a common gastropod species across a range of imaging conditions. By benchmarking five computer vision models against traditional laboratory techniques, we found that CounTR and Deep Data Space achieved a comparable accuracy to human counts, particularly when organisms are aggregated together. However, performance limitations emerged at greater organism densities, with most models plateauing around 400 individuals per image, underscoring the need for further refinement in handling high-density scenarios, which are common in many marine monitoring programmes. While machine learning techniques are not a substitute for traditional laboratory analyses, they offer a valuable complementary tool that can significantly reduce processing time and expand the spatio-temporal scope of ecological monitoring. The study also highlights the importance of standardized imaging protocols, careful model selection, and the need for further research into mixed-species and detritus-rich samples to improve model functionality. Future developments, including the integration of mobile imaging devices and citizen science initiatives, could further democratise and scale ecological data collection. As AI technologies continue to evolve, their thoughtful application holds great promise for transforming biodiversity monitoring and supporting more responsive and informed conservation strategies.

## Data Availability

“Data is provided within the manuscript or supplementary information files”.

## References

[CR1] Amini-Naieni, N., Amini-Naieni, K., Han, T., & Zisserman, A. (2023). Open-world text-specified object counting. *Cornell University*. 10.48550/arXiv.2306.01851

[CR2] Arje, J., Melvad, C., Jeppesen, M. R., Madsen, S. A., Raitoharju, J., Rasmussen, M. S., Losifidis, A., Tirronen, V., Gabbouj, M., Meissner, K., & Hoye, T. T. (2020). Automatic image-based identification and biomass estimation of invertebrates. *Methods in Ecology and Evolution,**11*, 922–931. 10.1111/2041-210X.13428

[CR3] Carion, N., Massa, F., Synnaeve, G., Usunier, N., Kirillov, A., & Zagoruyko, S. (2020). End-to-end object detection with transformers. European conference on computer vision,

[CR4] Catlin-Groves, C. L. (2012). The citizen science landscape: From volunteers to citizen sensors and beyond. *International Journal of Zoology*. 10.1155/2012/349630

[CR5] Chang, L., Yujie, Z., Andrew, R., & Weidi, X. (2022). *Countr: Transformer-based generalised visual counting* British Machine Vision Conference (BMVC),

[CR6] Costanza, R., d’Arge, R., De Groot, R., Farber, S., Grasso, M., Hannon, B., Limburg, K., Naeem, S., O’Neill, R. V., Paruelo, J., Raskin, R. G., Sutton, P., & van den Belt, M. (1997). The value of the world’s ecosystem services and natural capital. *Nature,**387*, 253–260. 10.1016/S0921-8009(98)00020-2

[CR7] Costanza, R., De Groot, R., Sutton, P., van der Ploeg, S., Anderson, S. J., Kubiszewski, I., Farber, S., & Turner, R. K. (2014). Changes in the global value of ecosystem services. *Global Environmnetal Change,**26*, 152–158. 10.1016/j.gloenvcha.2014.04.002

[CR8] Dafforn, K., Johnston, E., Ferguson, A., Humphrey, C., Monk, W., Nichols, S., Simpson, S., Tulbure, M., & Baird, D. (2015). Big data opportunities and challenges for assessing multiple stressors across scales in aquatic ecosystems. *Marine and Freshwater Research,**67*(4), 393–413. 10.1071/MF15108

[CR9] Dai, J., Qi, H., Xiong, Y., Zhang, G., Hu, H., & Wei, Y. (2017). Deformable convolutional networks. IEEE International Conference on Computer Vision,

[CR10] Danovaro, R., Carugati, L., Berzano, M., Cahill, A. E., Carvalho, S., Chenuil, A., Corinaldesi, C., Cristina, S., David, R., Dell’Anno, A., Dzhembekova, N., Garcés, E., Gasol, J. M., Goela, P., Féral, J.-P., Ferrera, I., Forster, R. M., Kurekin, A. A., Rastelli, E.,…Borja, A. (2016). Implementing and innovating marine monitoring approaches for assessing marine environmental status. *Frontiers in Marine Science*,* 3*(213). 10.3389/fmars.2016.00213

[CR11] Ditria, E. M., Lopez-Marcano, S., Sievers, M., Jinks, E. L., J., C., Brown, C. J., & Connolly, R. M. (2020). Automating the analysis of fish abundance using object detection: Optimizing animal ecology with deep learning. *Frontiers in Marine Science*,* 7*(429). 10.3389/fmars.2020.00429

[CR12] Dorkenwald, M., Barazani, N., Snoek, C. G. M., & Asano, Y. M. (2024). Pin: Positional insert unlocks object localisation abilities in vlms. *Proceedings of the IEEE/CVF Conference on Computer Vision and Pattern Recognition*, 13548–13558. 10.48550/arXiv.2402.08657

[CR13] Earp, H., Vye, S., Bohn, K., Burrows, M., Chenery, J., Dickens, S., Foster, C., Grist, H., Lamont, P., Long, S., Morrall, Z., Pocklington, J., Scott, A., Watson, G., West, V., Jenkins, S., Delany, J., & Sugden, H. (2022). Do you see what I see? Quantifying inter-observer variability in an intertidal marine citizen science experiment. *Citizen Science: Theory and Practice*. 10.5334/cstp.483

[CR14] Earp, H. S., & Liconti, A. (2020). Science for the future: The use of citizen science in marine research and conservation. YOUMARES 9-The Oceans: Our research, our future: Proceedings of the 2018 Conference for Young Marine Researcher, Oldenburg, Germany.

[CR15] Farjon, G., Huijun, L., & Edan, Y. (2023). Deep-learning-based counting methods, datasets, and applications in agriculture: A review. *Precision Agriculture*, *24*(5), 1683–1711. 10.48550/arXiv.2303.02632

[CR16] Fu, J., Liu, J., Tian, H., Li, Y., Bao, Y., Fang, Z., & Lu, H. (2019). Dual attention network for scene segmentation. IEEE/CVF Conference on Computer Vision and Pattern Recognition (CVPR),

[CR17] Gibert, A., Louty, F., Buscail, R., Baguette, M., Schatz, B., & Bertrand, J. A. M. (2022). Extracting quantitative information from images taken in the wild: A case study of two vicariants of the *Ophrys aveyronensis* species complex. *Diversity,**14*, Article 400. 10.3390/d14050400

[CR18] Goldman, E., Herzig, R., Eisenschtat, A., Ratzon, O., Levi, I., Goldberger, J., & Hassner, T. (2019). Precise detection in densely packed scenes. *Proceedings of the IEEE/CVF Conference on Computer Vision and Pattern Recognition*, 5277–5236. 10.48550/arXiv.1904.00853

[CR19] Halpern, B. S., Walbridge, S., Selkoe, K. A., Kappel, C. V., Micheli, F., D’Agrosa, C., Bruno, J. F., Casey, K. S., Ebert, C., Fox, H. E., Fujita, R., Heinemann, D., Lenihan, H. S., Madin, E. M. P., Perry, M. T., Selig, E. R., Spalding, M., Steneck, R., & Watson, R. (2008). A global map of human impact on marine ecosystems. *Science,**319*, 948–952. 10.1126/science.1149345 18276889 10.1126/science.1149345

[CR20] Høye, T. T., Dyrmann, M., Kjær, C., Nielsen, J., Bruus, M., Mielec, C. L., Vesterdal, M. S., Bjerge, K., Madsen, S. A., Jeppesen, M. R., & Melvad, C. (2022). Accurate image-based identification of macroinvertebrate specimens using deep learning—How much training data is needed? *PeerJ,**10*, Article e13837. 10.7717/peerj.13837 36032940 10.7717/peerj.13837 PMC9415355

[CR21] Hsieh, M. R., Lin, Y. L., & Hsu, W. H. (2017). Drone-based object counting by spatially regularized regional proposal network. *Proceedings of the IEEE international conference on computer vision*, 4145–4153. 10.48550/arXiv.1707.05972

[CR22] Hu, J., Shen, L., & Sun, G. (2018). Squeeze-and-excitation networks. *Proceedings of the IEEE conference on computer vision and pattern recognition*, 7132–7141. 10.1109/CVPR.2018.00745

[CR23] Jaballah, S., Garcia, G. F., Martignac, F., Parisey, N., Jumel, S., Roussel, J.-M., & Dézerald, O. (2023). A deep learning approach to detect and identify live freshwater macroinvertebrates. *Aquatic Ecology,**57*, 933–949. 10.1007/s10452-023-10053-7

[CR24] The jamovi project. (2022). *jamovi (version 2.5) [computer software]*. https://www.jamovi.org

[CR25] Jiang, Q., Li, F., Zeng, Z., Ren, T., Liu, S., & Zhang, L. (2024). T-rex2: Towards generic object detection via text-visual prompt synergy. *Cornell University*. 10.48550/arXiv.2403.14610

[CR26] Khan, S., Naseer, M., Hayat, M., Zamir, S. W., Khan, F. S., & Shah, M. (2022). Transformers in vision: A survey. *ACM Computing Surveys (CSUR),**54*(10s), 1–41. 10.1145/350524

[CR27] Langenkamper, D., Simon-Lledo, E., Hosking, B., Jones, D. O. B., & Nattkemper, T. W. (2019). On the impact of citizen science-derived data quality on deep learning based classification in marine images. *PLoS ONE,**14*(6), Article e0218086. 10.1371/journal.pone.0218086 31188894 10.1371/journal.pone.0218086 PMC6561570

[CR28] Liu, S., Zeng, Z., Ren, T., Li, F., Zhang, H., Yang, J., Jiang, Q., Li, C., Yang, J., Su, H., Zhu, J., & Zhang, L. (2023). Grounding dino: Marrying dino with grounded pre-training for open-set object detection. *Cornell University*. 10.48550/arXiv.2303.05499

[CR29] Ma, Z., Hong, X., & Shangguan, Q. (2023). Can SAM count anything? An empirical study on SAM counting. *Cornell University*. 10.48550/arXiv.2304.10817

[CR30] Mattingly, M., Barnas, A., Ellis-Felege, S., Newman, R., Iles, D., & Desell, T. (2016). Developing a citizen science webportal for manual and automated ecological image detection. 2016 IEEE 12th International Conference on e-Science (e-Science),

[CR31] Moolna, A., Duddy, M., Fitch, B., & White, K. (2020). Citizen science and aquatic macroinvertebrates: Public engagement for catchment-scale pollution vigilance. *Ecoscience,**27*(4), 303–317. 10.1080/11956860.2020.1812922

[CR32] Pinho, C., Kaliontzopoulou, A., Ferreira, C. A., & Gama, J. (2022). Identification of morphologically cryptic species with computer vision models: Wall lizards (squamata: Lacertidae: *Podarcis*) as a case study. *Zoological Journal of the Linnean Society,**198*(1), 184–201. 10.1093/zoolinnean/zlac087/6777764

[CR33] Poloczanska, E., Brown, C. J., Sydeman, W. J., Kiessling, W., Schoeman, D. S., Moore, P., Brander, K., Bruno, J. F., Buckley, L. B., Burrows, M. T., Duarte, C. M., Halpern, B. S., Holding, J., Kappel, C. V., O’Connor, M. I., Pandolfi, J. M., Parmesan, C., Schwing, F., Thompson, S. A., & Richardson, A. J. (2013). Global imprint of climate change on marine life. *Nature Climate Change,**3*, 919–925. 10.1038/nclimate1958

[CR34] Ranjan, V., Sharma, U., Nguyen, T., & Haoai, M. (2021). Learning to count everything. *Proceedings of the IEEE/CVF Conference on Computer Vision and Pattern Recognition*, 3394–3403. 10.48550/arXiv.2104.08391

[CR35] Ren, T., Chen, Y., Jiang, Q., Zeng, Z., Xiong, Y., Liu, W., Ma, Z., Shen, J., Goao, Y., Jiang, X., Chen, X., Song, Z., Zhang, Y., Huang, H. M., Gao, H., Liu, S., Zhang, H., Li, F., Yu, K., & Zhang, L. (2024). *Dino-x: A unified vision model for open-world object detection and understanding*. C. University.

[CR36] Robinson, D., Delany, J., & Sugden, H. (2024). Beyond science: Exploring the value of co-created citizen science for diverse community groups. *Citizen Science: Theory and Practice,**9*(1), 1–13. 10.5334/cstp.682

[CR37] Schneider, C. A., Rasband, W. S., & Eliceiri, K. W. (2012). NIH image to ImageJ: 25 years of image analysis. *Nature Methods,**9*(7), 671–675. 10.1038/nmeth.2089 22930834 10.1038/nmeth.2089 PMC5554542

[CR38] Shi, M., Lu, H., Feng, C., Liu, C., & Cao, Z. (2022). Represent, compare, and learn: A similarity-aware framework for class-agnostic counting. *Proceedings of the IEEE/CVF Conference on Computer Vision and Pattern Recognition*, 9529–9538. 10.48550/arXiv.2203.08354

[CR39] Shi, Z., Sun, Y., & Zhang, M. (2024). Training-free object counting with prompts. *Proceedings of the IEEE/CVF Winter Conference on Applications of Computer Vision*, 323–331. 10.48550/arXiv.2307.00038

[CR40] Siddiqui, M. I., Sheikh, M. U., Abid, H., & Khan, M. H. (2024). Persense: Personalized instance segmentation in dense images. *Cornell University*. 10.48550/arXiv.2405.13518

[CR41] Singh, T., Gangloff, H., & M-T., P. (2023). Object counting from aerial remote sensing images: Application to wildlife and marine mammals. 2023 IEEE International Geoscience and Remote Sensing Symposium, Pasadena, United States.

[CR42] Sola, J. C. (1996). Population dynamics, reproduction, growth, and secondary production of the mud-snail *Hydrobia ulvae* (Pennant). *Journal of Experimental Marine Biology and Ecology,**205*(1–2), 49–62. 10.1016/S0022-0981(96)02597-X

[CR43] Tarling, P., Cantor, M., Clap´es, A., & Escalera, S. (2022). Deep learning with self-supervision and uncertainty regularization to count fish in underwater images. *PLoS ONE*, *17*(5), e0267759. 10.1371/journal.pone.0267759 10.1371/journal.pone.0267759 PMC9067705 35507631

[CR44] Terry, J. C. D., Roy, H. E., & August, T. A. (2020). Thinking like a naturalist: Enhancing computer vision of citizen science images by harnessing contextual data. *Methods in Ecology and Evolution,**11*(2), 303–315. 10.1111/2041-210X.13335

[CR45] Vaswani, A., Shazeer, N., Parmar, N., Uszkoreit, J., Jones, L., Gomez, A. N., Kaiser, L., & Polosukhin, I. (2017). Attention is All you Need. In I. Guyon, U. Von Luxburg, S. Bengio, H. Wallach, R. Fergus, S. Vishwanathan, & R. Garnett, NeurIPS Proceedings,

[CR46] Wang, Q., Wu, T., Zheng, H., & Guo, G. (2020). Hierarchical pyramid diverse attention networks for face recognition. *Proceedings of the IEEE/CVF Conference on Computer Vision and Pattern Recognition*, 8326–8335. 10.1109/CVPR42600.2020.00835

[CR47] Wei, X. Y., Zhang, L., Ma, H. Y., & Zhang, X.-F. (2024). SCU-counting: A large-scale benchmark dataset for multi-class object counting. *Transportation Research Part C*, *163*(104608). 10.1016/j.trc.2024.104608

[CR48] Weinstein, B. G. (2017). A computer vision for animal ecology. *Journal of Animal Ecology,**87*(3), 533–545. 10.1111/1365-2656.12780 29111567 10.1111/1365-2656.12780

[CR49] Wernberg, T., Thomsen, M. S., Baum, J. K., Bishop, M. J., Bruno, J., F,, Coleman, M. A., Filbee-Dexter, K., Gagnon, K., He, Q., Murdiyarso, D., Rogers, K., Silliman, B. R., Smale, D. A., Starko, S., & Vanderklift, M. A. (2024). Impacts of climate change on marine foundation species. *Annual Review of Marine Science*, *16*, 247–282. 10.1146/annurev-marine-042023-093037 10.1146/annurev-marine-042023-093037 37683273

[CR50] Willi, M., Pitman, R. T., Cardoso, A. W., Locke, C., Swanson, A., Boyer, A., Veldthuis, M., & Fortson, L. (2018). Identifying animal species in cam- era trap images using deep learning and citizen science. *Methods in Ecology and Evolution,**10*(1), 80–91. 10.1111/2041-210X.13099

[CR51] Woo, S., Park, J., Lee, J. Y., & Kweon, S. (2018). Cbam: Convolutional block attention module. *Proceedings of the European conference on computer vision (ECCV)*, 3–19. 10.48550/arXiv.1807.06521

[CR52] Worm, B., Barbier, E. B., Beaumont, N., Duffy, J. E., Folke, C., Halpern, B. S., Jackson, J. B. C., Lotze, H. K., Micheli, F., Palumbi, S. R., Sala, E., Selkoe, K. A., Stachowicz, J. J., & Watson, R. (2006). Impacts of biodiversity loss on ocean ecosystem services. *Science,**314*(5800), 787–790. 10.1126/science.1132294 17082450 10.1126/science.1132294

[CR53] Xu, L., Bennamoun, M., An, S., Sohel, F., & Boussaid, F. (2019). Deep learning for marine species recognition. In V. E. Bales, S. S. Roy, D. Sharma, & P. Samui (Eds.), *Handbook of deep learning applications* (Vol. Vol. 136, pp. 129–145). Springer Science + Business Media. 10.1007/978-3-030-11479-4_7

[CR54] Yang, J., Ren, P., Zhang, D., Chen, D., Wen, F., Li, H., & Hua, G. (2017). Neural aggregation network for video face recognition. *Proceedings of the IEEE conference on computer vision and pattern recognition*, 4362–4371. 10.48550/arXiv.1603.05474

[CR55] Yuan, Y., Huang, L., Guo, J., Zhang, C., Chen, X., & Wang, J. (2018). Object context network for scene parsing. *Cornell University*. 10.48550/arXiv.1809.00916

[CR56] Zare, M., Akbarialiabad, H., Parsaei, H., Asgari, Q., Alinejad, A., Bahreini, M. S., Hosseini, S. H., Ghofrani-Jahromi, M., Shahriarirad, R., Amirmoezzi, Y., Shahriarirad, S., Zeighami, A., & Abdollahifard, G. (2022). A machine learning-based system for detecting leishmaniasis in microscopic images. *BMC Infectious Diseases,**22*(1), 48. 10.1186/s12879-022-07029-7 35022031 10.1186/s12879-022-07029-7 PMC8754077

